# Supplementary material for: Right Cortical Infarction and a Reduction in Putamen Volume May Be Correlated with Empathy in Patients after Subacute Ischemic Stroke—A Multimodal Magnetic Resonance Imaging Study
Source: J Clin Med. 2022 Jul 31;11(15):4479. doi: 10.3390/jcm11154479 (PMC9369598; doi:10.3390/jcm11154479)

Supplemental Data: Putaminal volumes of quantitative images and corresponding result of EQ for each patient

Subject 1 Putamen volume = 10.3ml EQ = 19

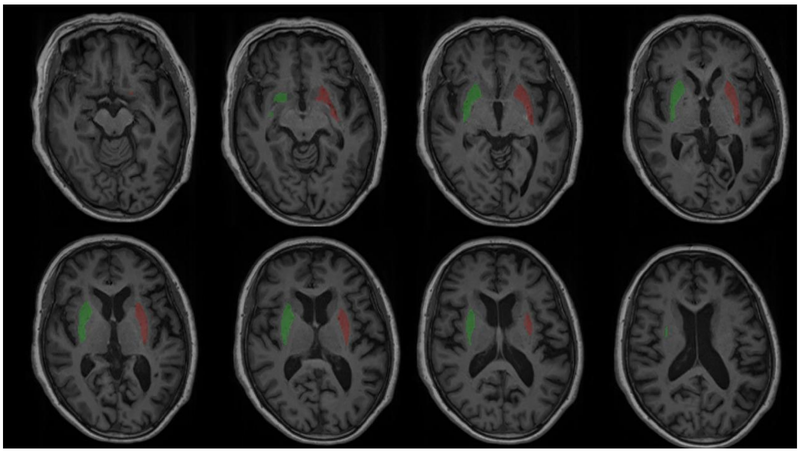

Subject 2 Putamen volume = 9.92ml EQ = 52

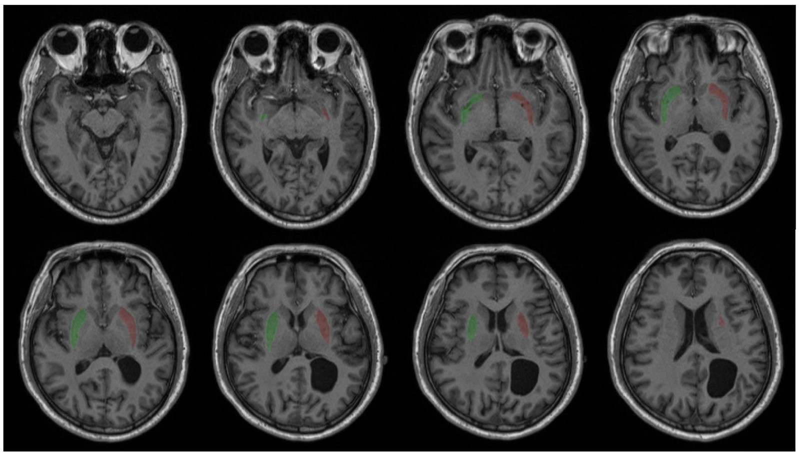

Subject 3 Putamen volume = 11.6ml EQ = 57

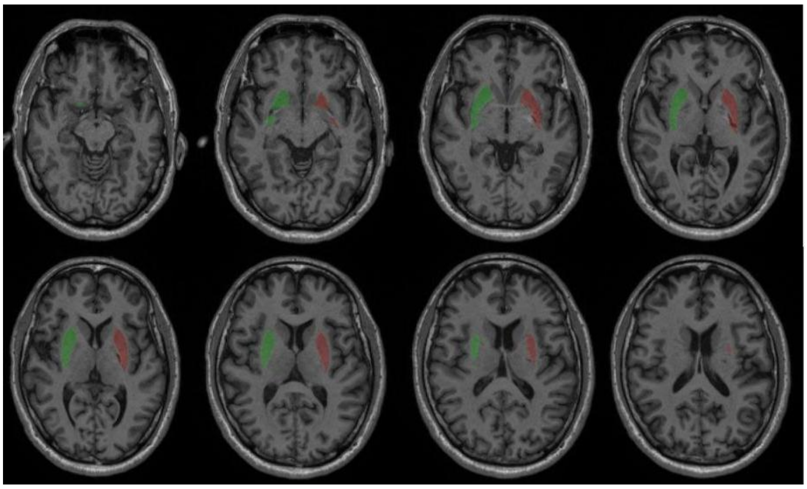

Subject 4 Putamen volume = 11.2ml EQ = 51

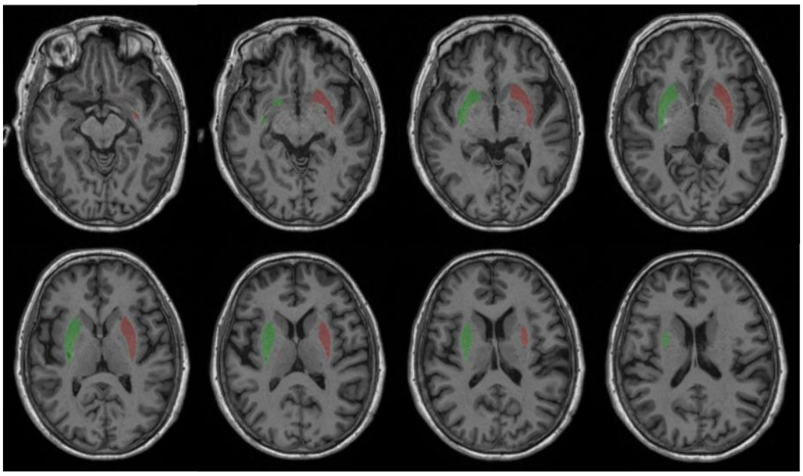

Subject 5 Putamen volume =10.6ml EQ = 60

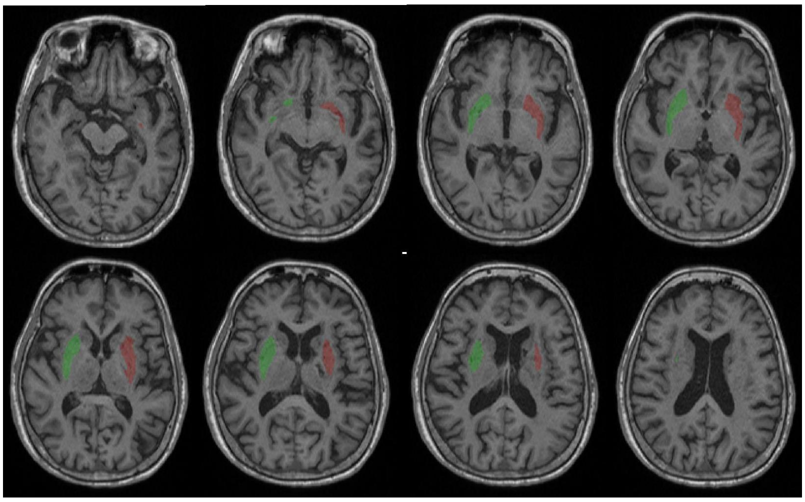

Subject 6 Putamen volume = 11.6ml EQ = 73

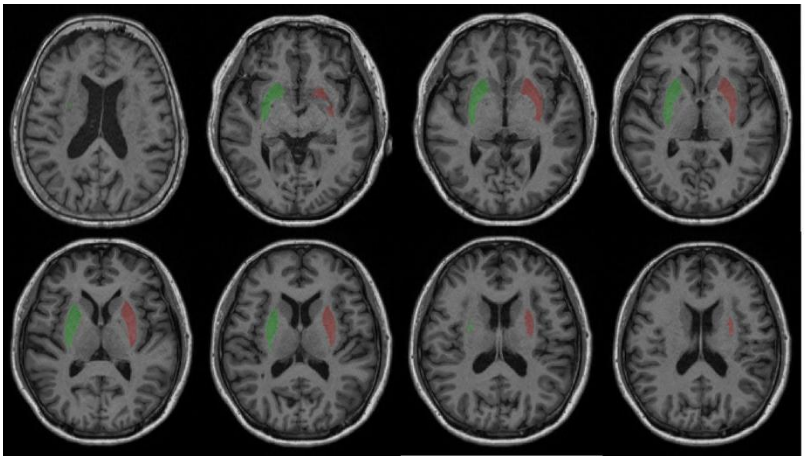

Subject 7 Putamen volume = 10.9ml EQ = 44

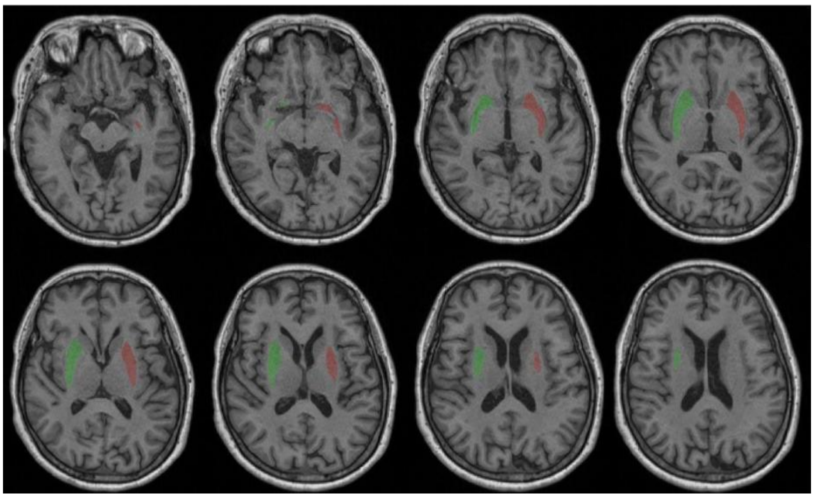

Subject 8 Putamen volume = 10.2ml EQ = 31

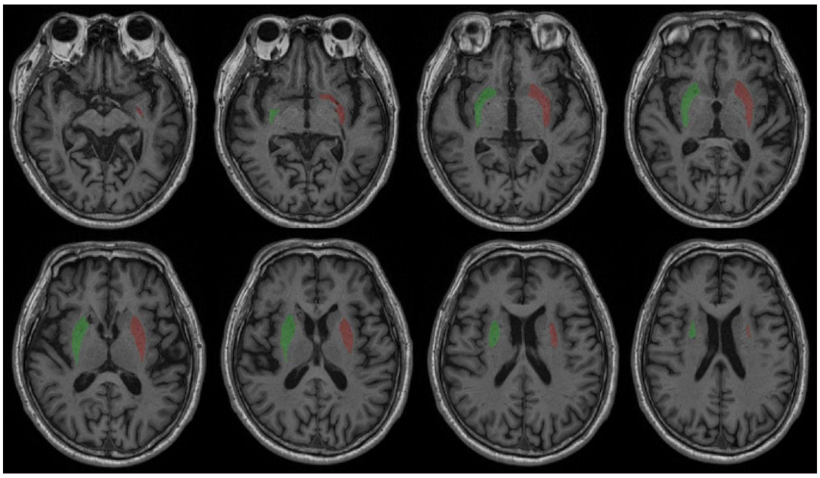

Subject 9 Putamen volume = 10.1ml EQ = 50

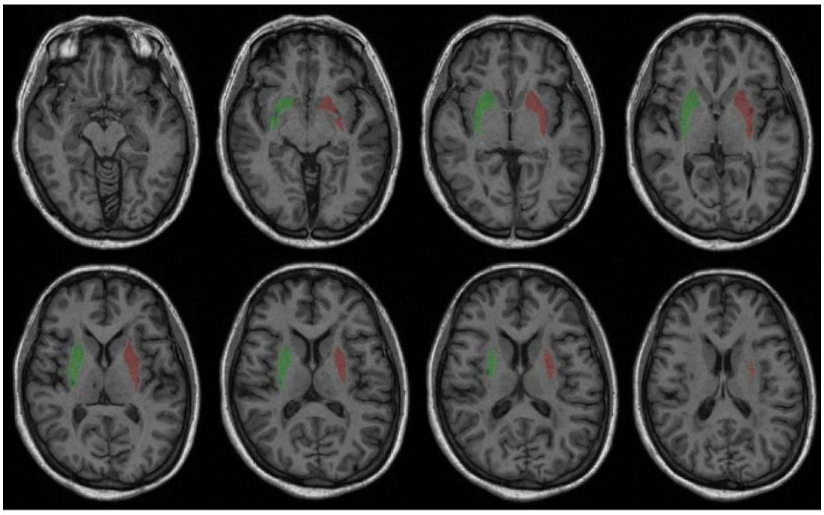

Subject 10 Putamen volume = 11.7ml EQ = 57

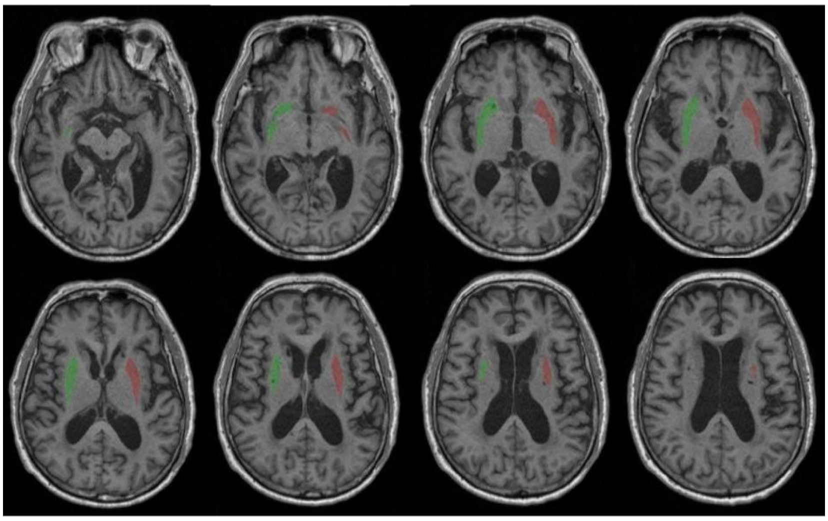

Subject 11 Putamen volume = 10ml EQ = 47

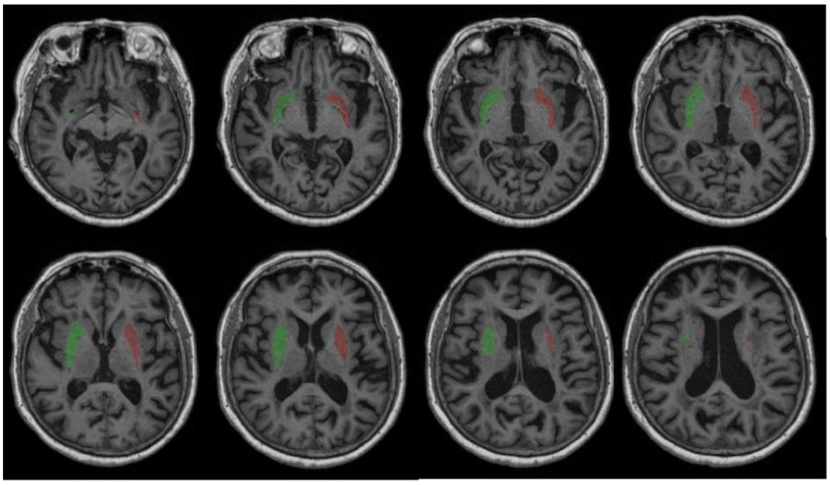

Subject 12 Putamen volume = 10ml EQ = 33

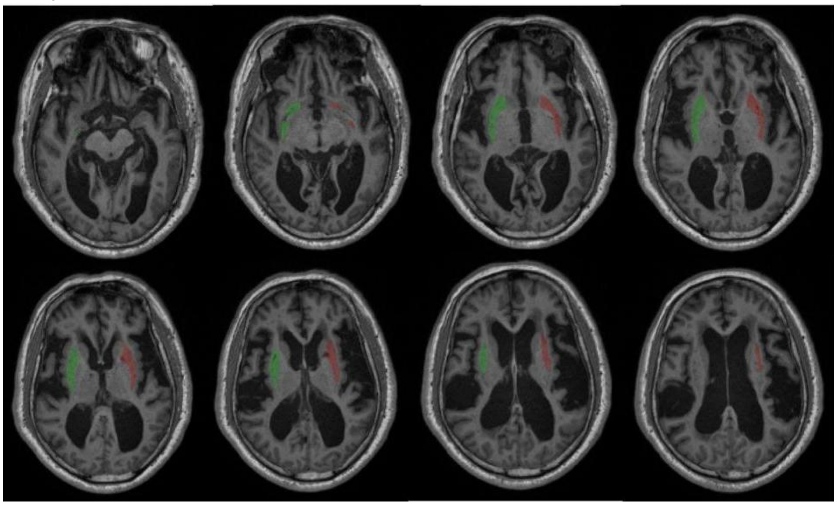

Subject 13 Putamen volume = 10.3ml EQ = 24

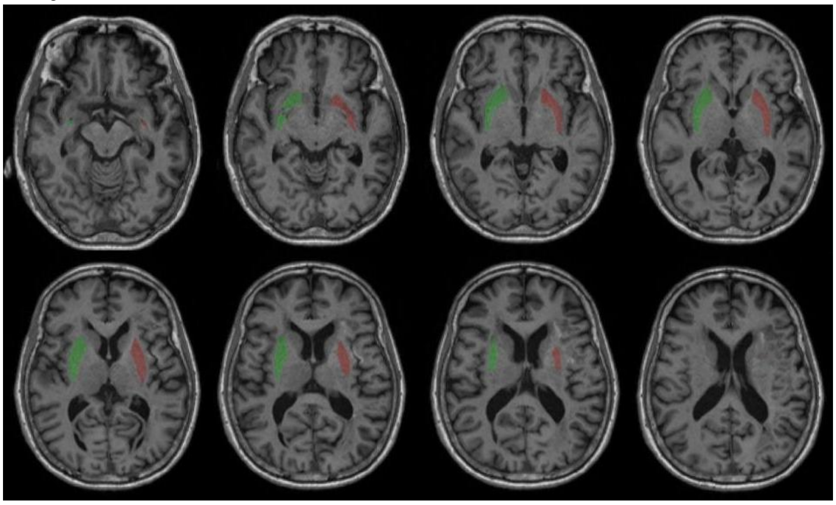

Subject 14 Putamen volume = 8.1ml EQ = 20

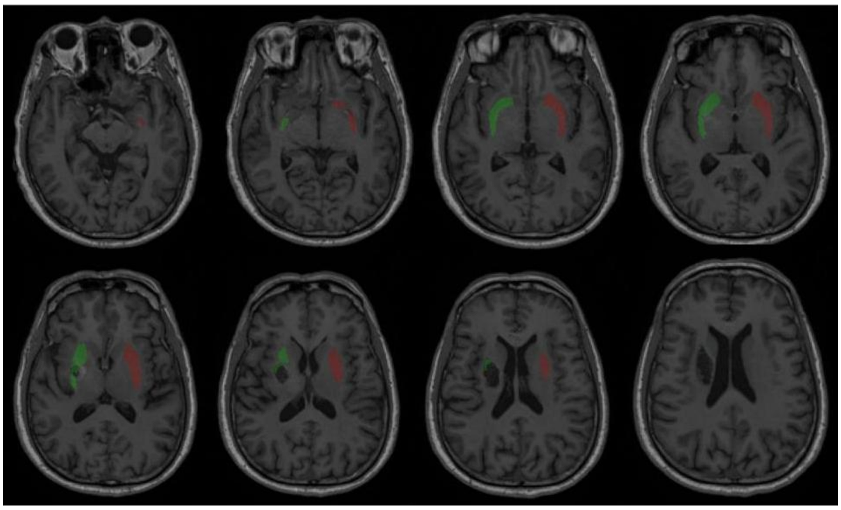

Subject 15 Putamen volume = 11.3ml EQ = 30

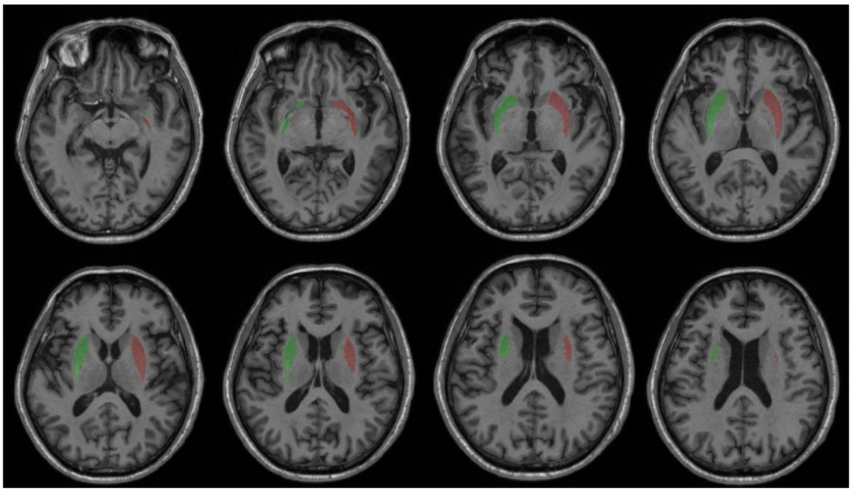

Subject 16 Putamen volume = 11.8ml EQ = 33

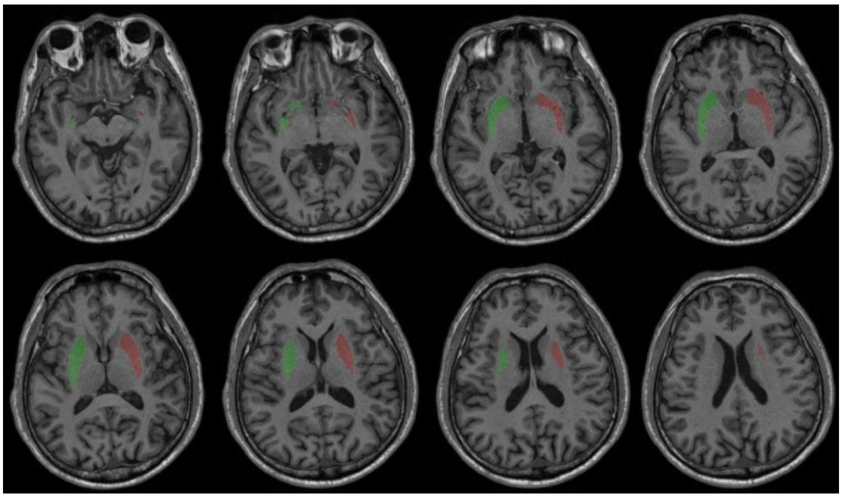

Subject 17 Putamen volume = 10.2ml EQ = 31

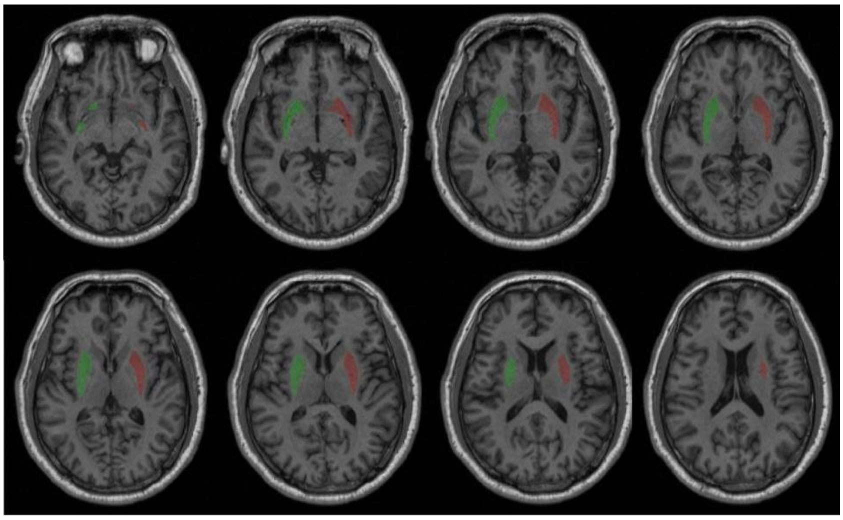

Subject 18 Putamen volume = 10.7ml EQ = 36

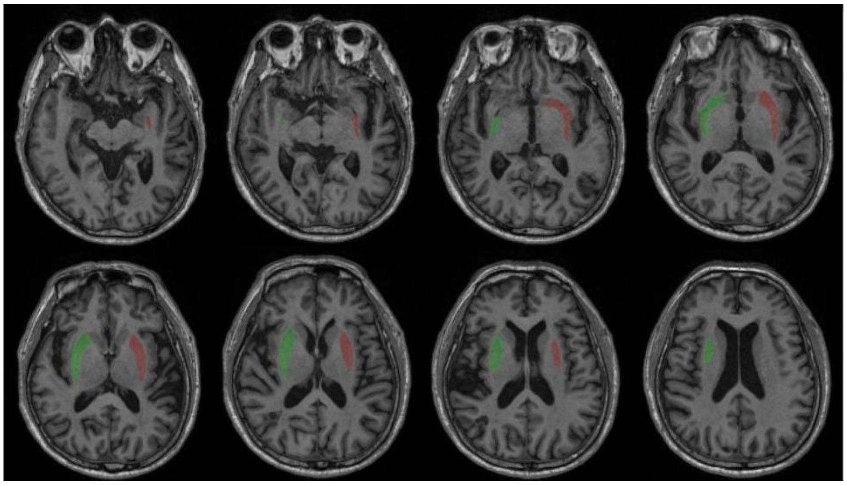

Subject 19 Putamen volume = 10.6ml EQ = 50

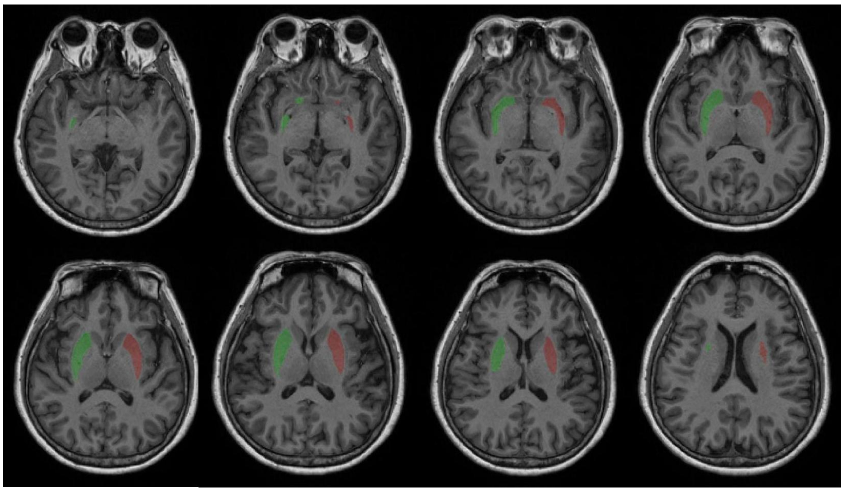

Subject 20 Putamen volume = 10ml EQ = 45

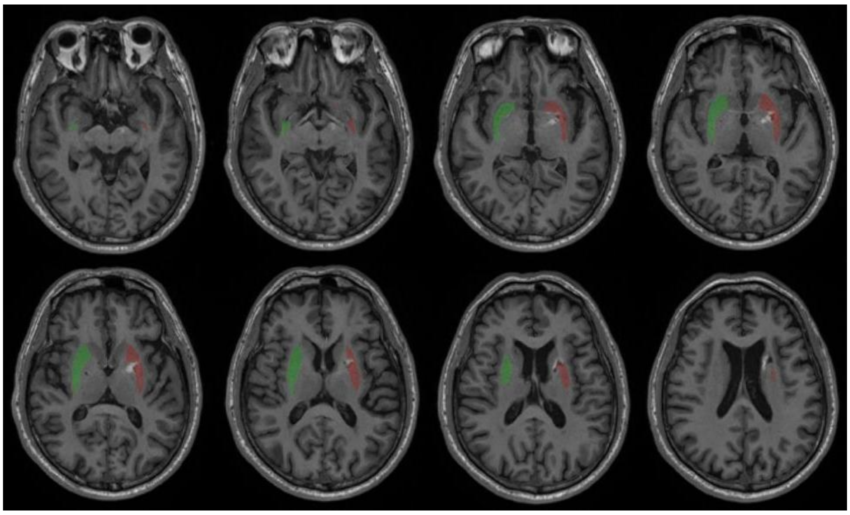

Subject 21 Putamen volume = 11.4ml EQ = 39

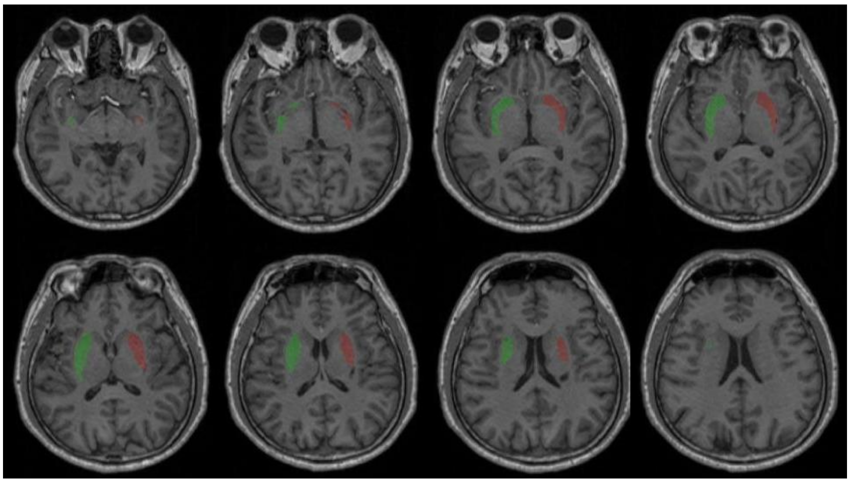

Subject 22 Putamen volume = 8.73ml EQ = 38

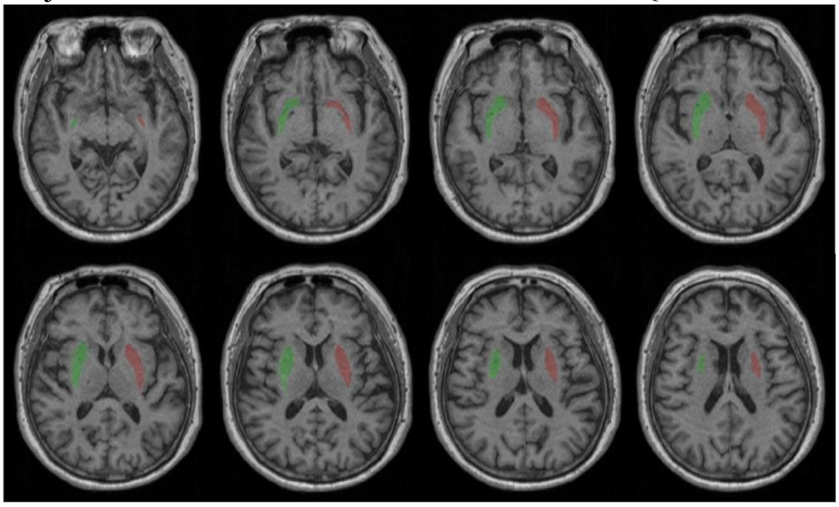

Subject 23 Putamen volume = 10.8ml EQ = 39

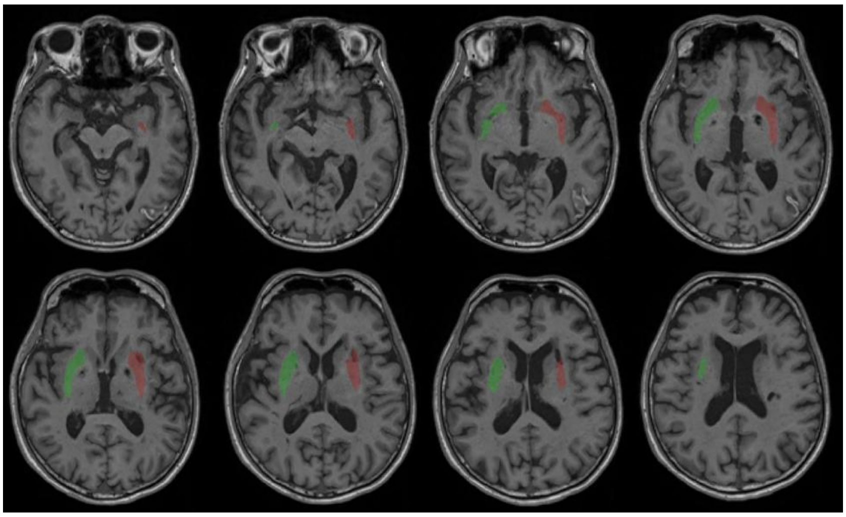

Subject 24 Putamen volume = 5.53ml EQ = 23

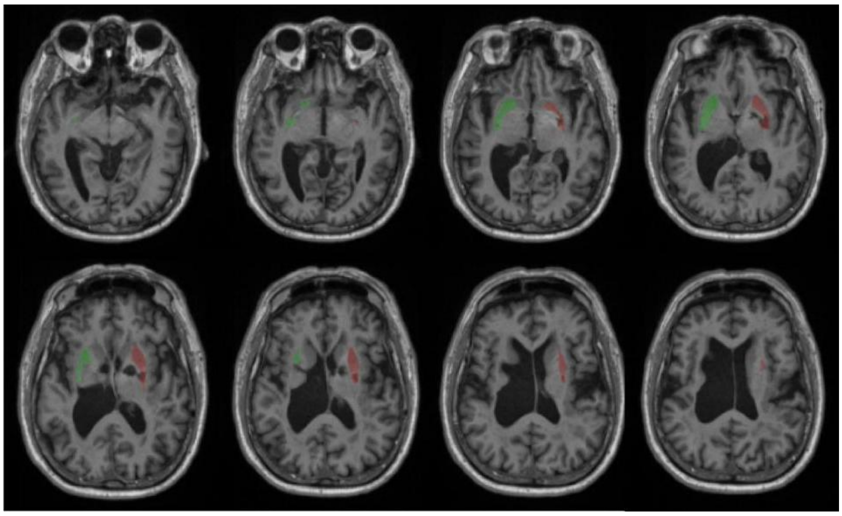

Subject 25 Putamen volume = 10.9ml EQ = 46

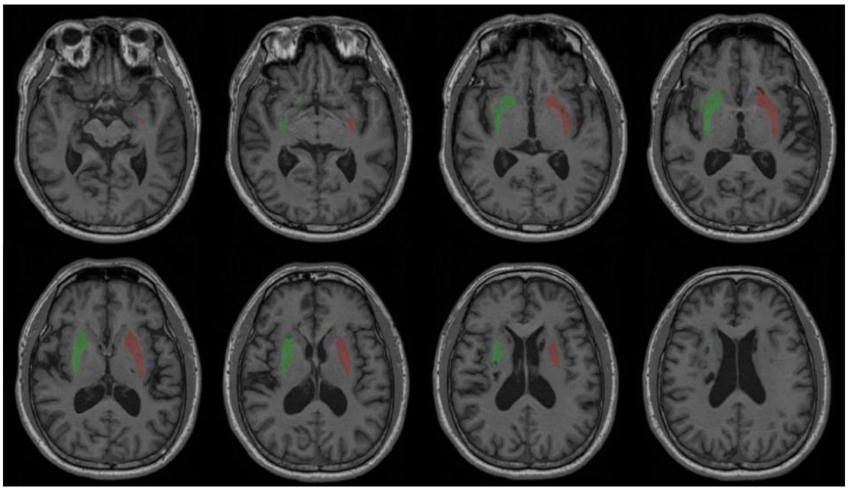

Subject 26 Putamen volume = 12.9ml EQ = 20

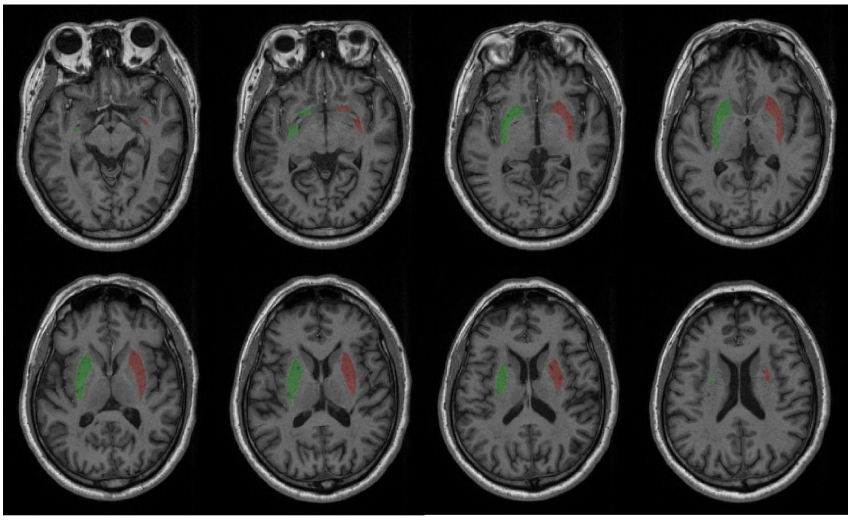

Subject 27 Putamen volume = 10.5ml EQ = 48

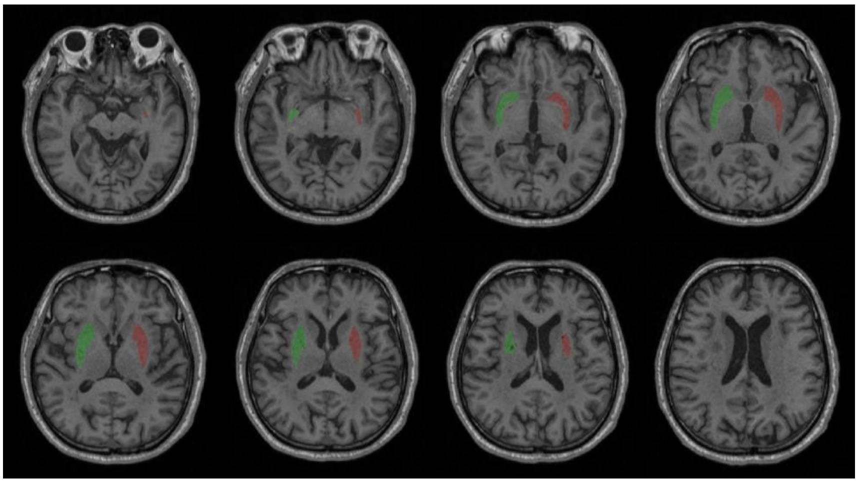

Subject 28 Putamen volume = 10.8ml EQ = 52

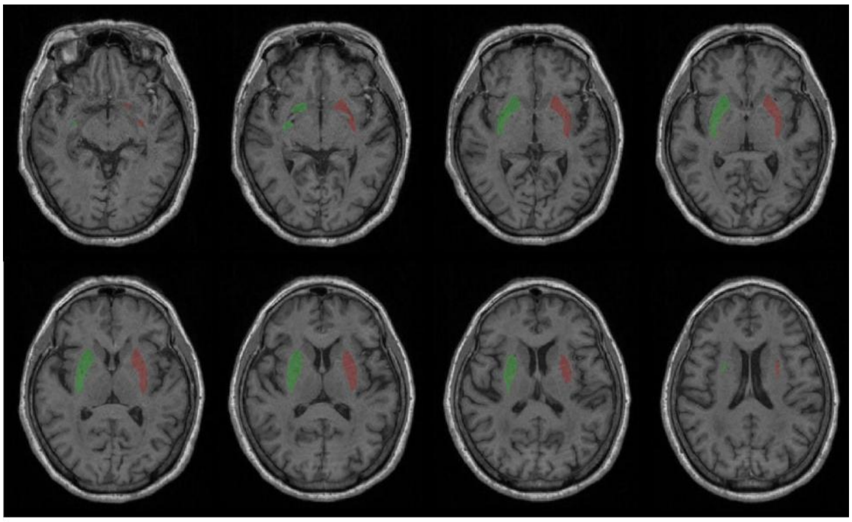

Subject 29 Putamen volume = 10.7ml EQ = 26

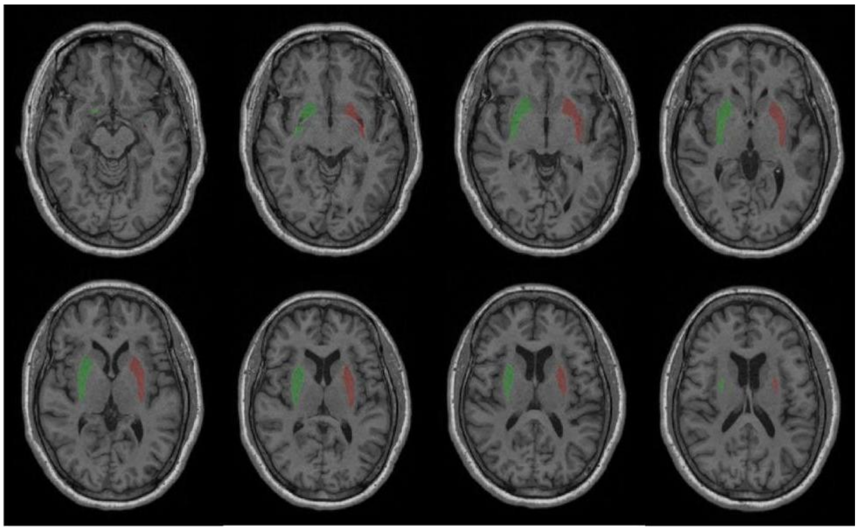

Subject 30 Putamen volume = 10.3ml EQ = 16

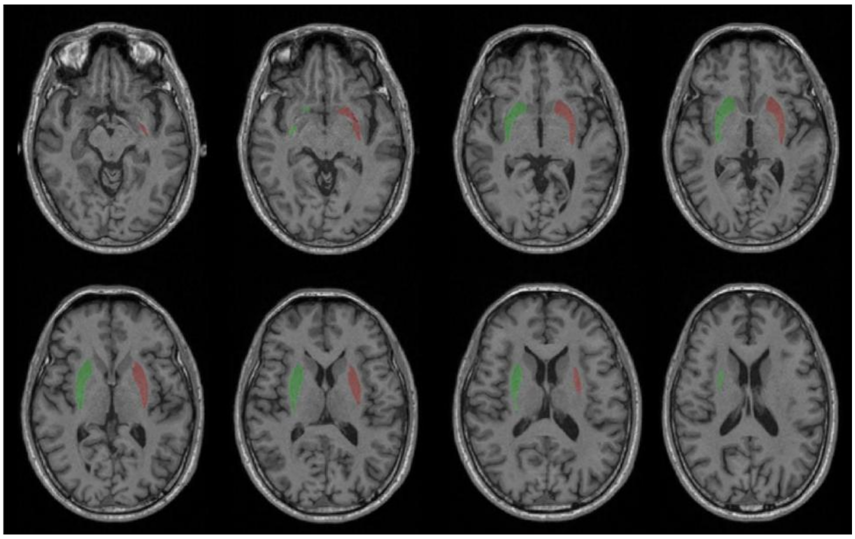

Subject 31 Putamen volume = 12.7ml EQ = 36

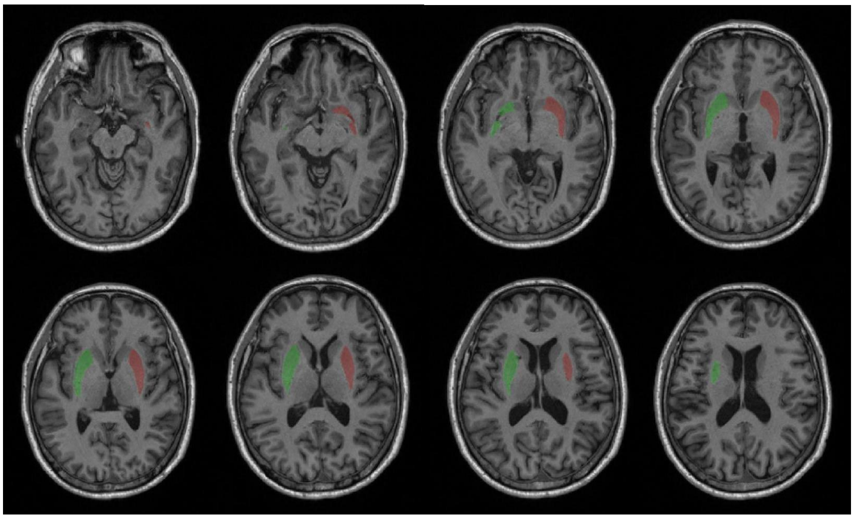

Subject 32 Putamen volume = 13ml EQ = 33

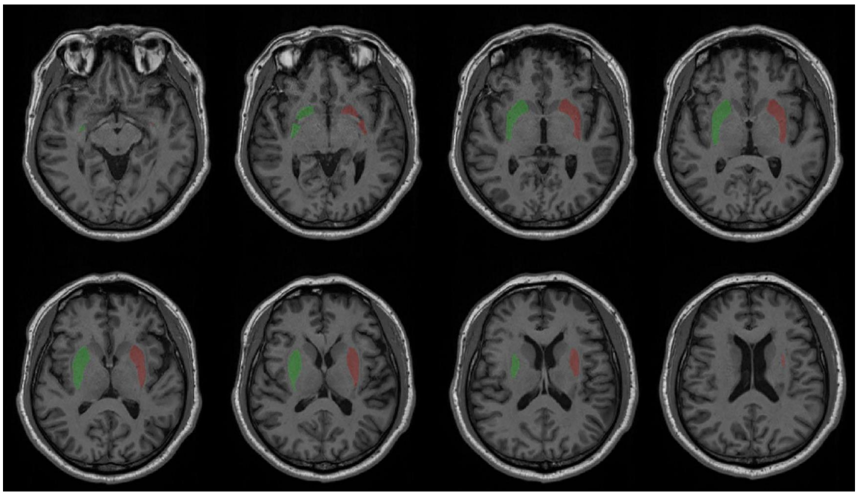

Subject 33 Putamen volume = 8.72ml EQ = 35

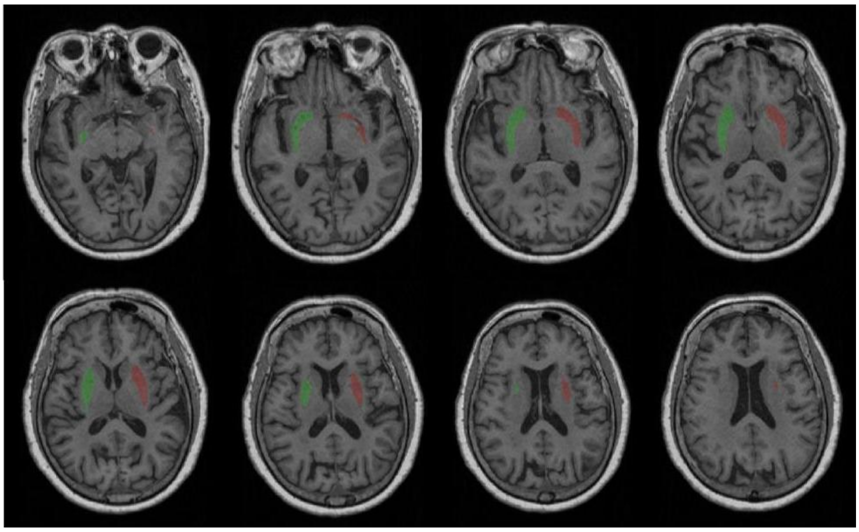

Subject 34 Putamen volume = 8.95ml EQ = 32

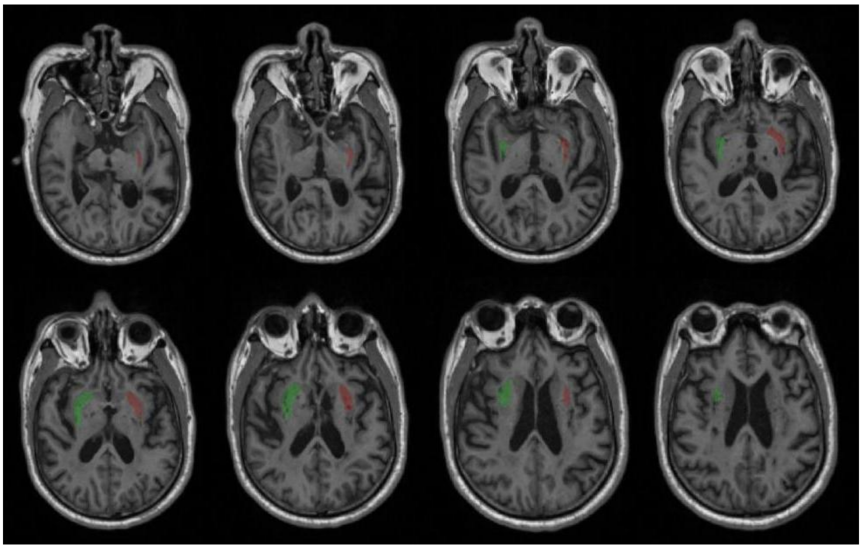

Subject 35 Putamen volume = 8.75ml EQ = 34

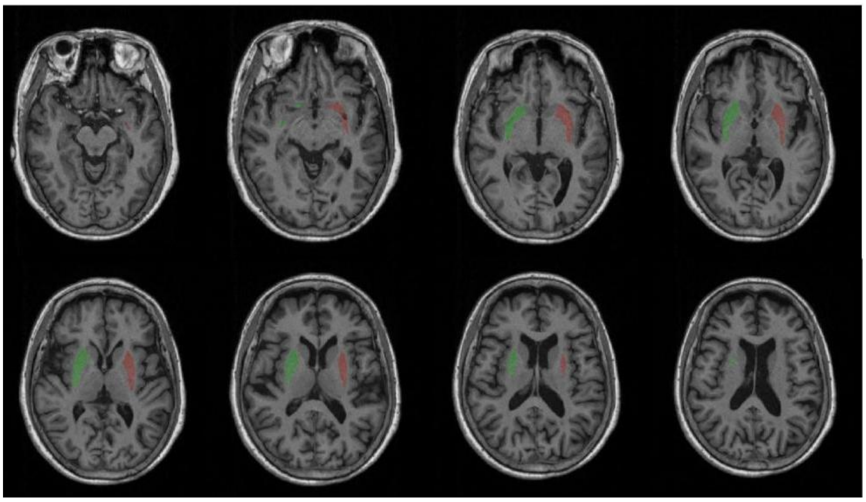

Subject 36 Putamen volume = 10.8ml EQ = 51

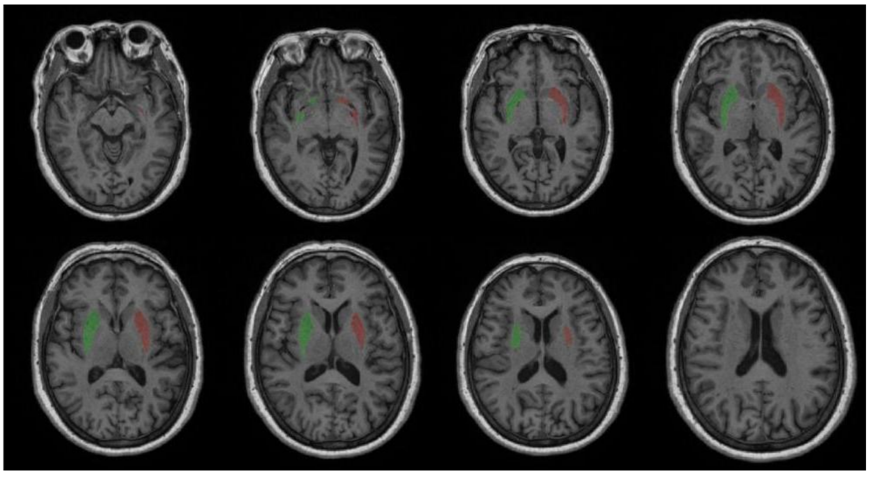

Subject 37 Putamen volume = 8.96ml EQ = 24

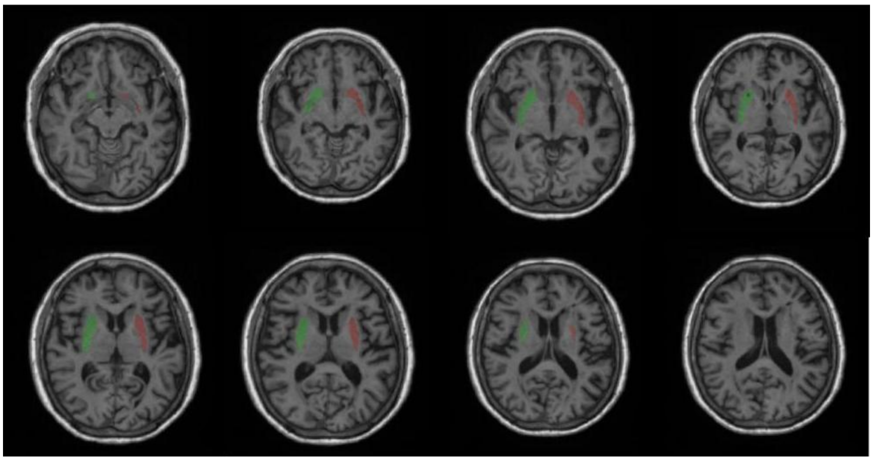

Subject 38 Putamen volume = 9.24ml EQ = 43

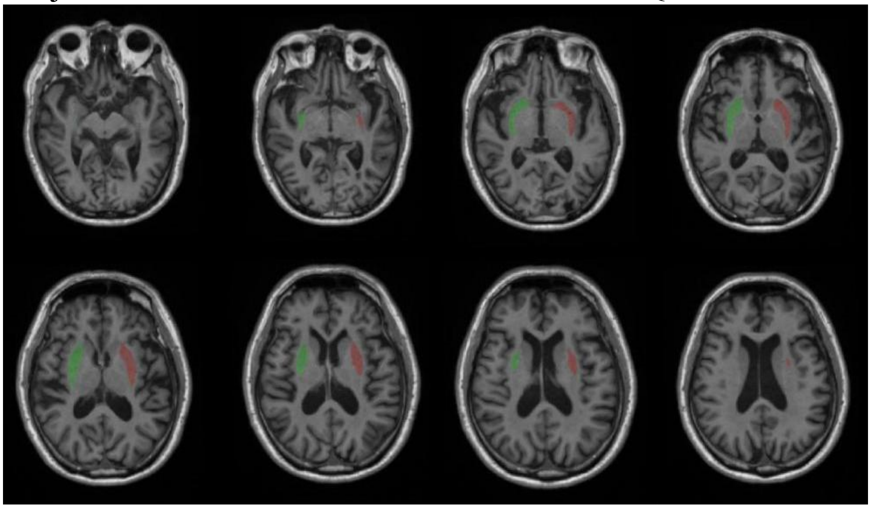

Subject 39 Putamen volume = 12ml EQ = 45

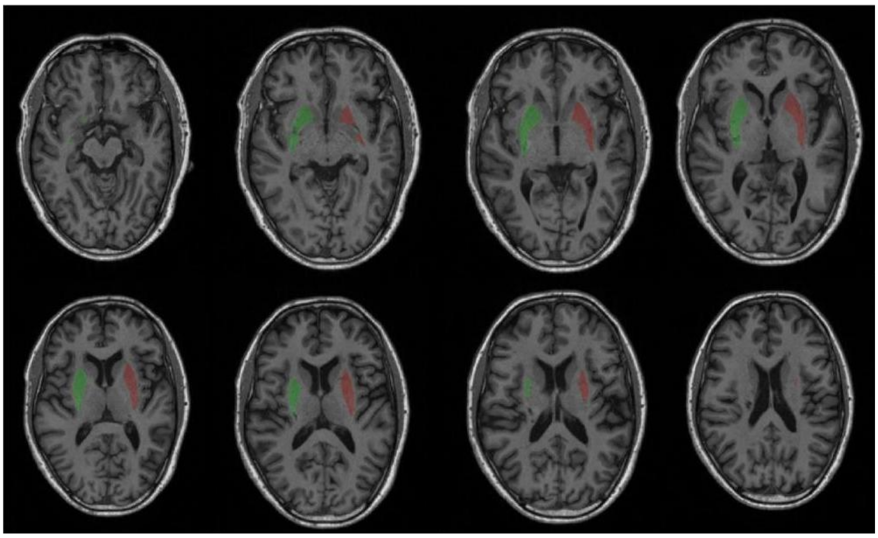

Subject 40 Putamen volume = 8.38ml EQ = 20

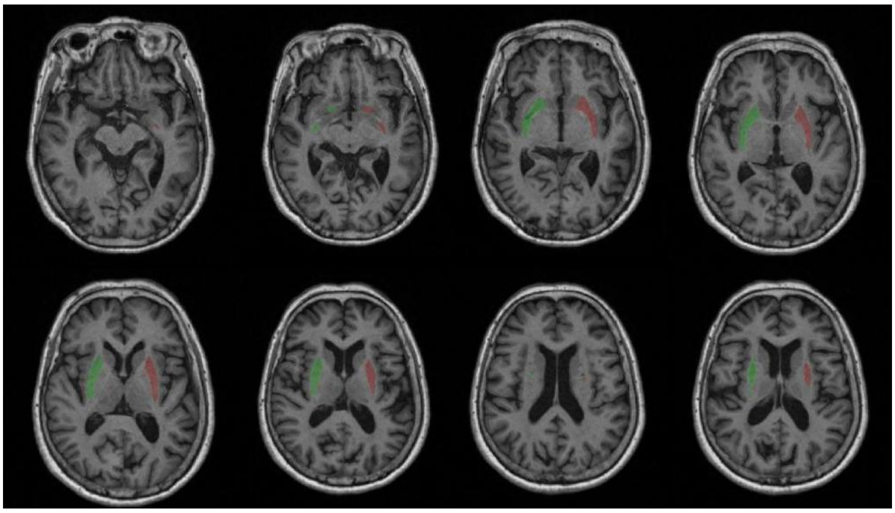

Subject 41 Putamen volume = 11.1ml EQ = 37

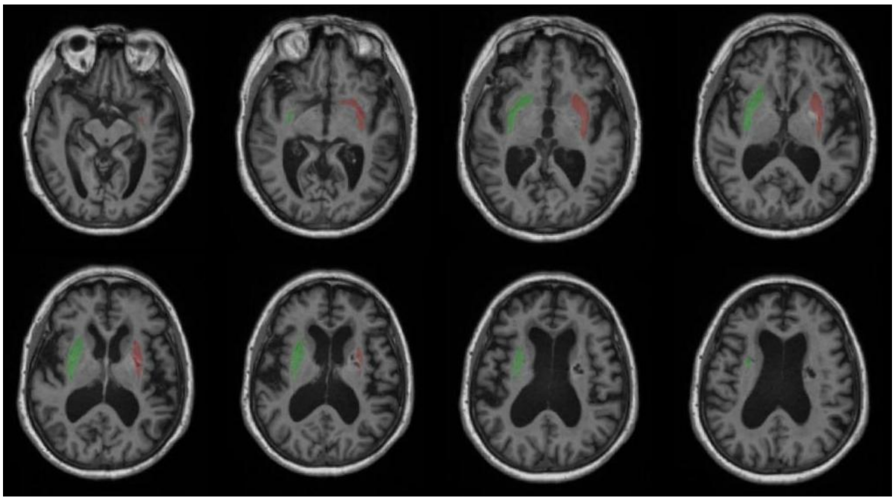

Supplement: Supplementary file 1 [file jcm-11-04479-s001.zip › jcm-1784824-supplementary.pdf]
